# Scarpa's Observations on Several Diseases of the Eyes, Extracted from His "Saggio di Osservazione et di Esperienze sulle malattie degli occhi, &c." Pavia, 1801

**Published:** 1803-12-01

**Authors:** 


### Scarpa's Observations on several Diseases of the Eyes, *extracted from his "Saggio di Osservazione et di Esperienze sulle malattie degli occhi, &c." Pavia,* 1801.

### *On the puriform Running of the Eye-lids and on the Fistula Lacrymalis.*

Mr. Scarpa distinguishes the last from the former disease in the following manner: Fistula lacrymalis, he calls that state of the saccus lacrymalis, when it is much extended, exulcerated, spongy, corroded, and sometimes attended with caries of the os unguis. The puriform running of the eye-lids takes place, if on a pressure of the sound lacrymal sac, a puriform, granulous, and yellowish matter issues through the puncta lacrymalia. This matter is not generated in the sac, but originates from the internal surface of the eye-lids, particularly of the lower ones, and is likewise secreted by the glandulæ meibomianæ.

This affection may be divided into four periods: 1. The matter is collecting itself in the sac, but finds an easy passage through the nose. 2. The passage being obstructed more or less, it begins to extend the sac. 3. The saccus lacrymalis is inflamed, corroded, and an ulceration takes place (fistula lacrymalis). 4. Caries of the os unguis supervenes. The cure of this affection differs according to the just stated periods. In the first, it is advisable to apply stimulant remedies, viz. the unguent. jannin. a diluted solution of vitriol. alb. The excoriations of the eye-lids are best removed by the unguentum citrinum Edinb. or by the cautious application of lapis infernalis. At the same time, aqu. plantaginis, with a little spirit of wine, must be injected into the puncta lacrymalia, till it has come into the sac. In the second period, an incision is made beneath the white macula, where the tendo and the ligament of the musc. orbicularis is situated, along the saccus lacrymalis, and a probe introduced into the nose through the canalis nasalis. The probe being taken out, a bougie of resina elastica is to be introduced; the cavity of the sac must be filled with lint. On renewing the dressing an ointment of red precipitate and gum arabic is applied to the lint, or the whole cavity is filled with red precipitate or alum, and the eye treated as in the former period. As soon as the sac has recovered its natural size, the lint is moistened with lime water and mel rosarum; and in lieu of the bougie, a small leaden tube is introduced, which being, after some time, extracted, the opening is suffered to close. When the disease is arrived at the third period, the sac generally opens itself at an unfavourable place, on which account it is necessary to make an incision at the above indicated place, and to treat the wound in the same manner as before mentioned. In the fourth period, the sac is either found entire, but the os unguis internally corroded, or the sac is open and the os unguis carious. In the first case the sac must be opened and cleaned, by which the bone will be exfoliated and the sac soon be closed. In the second case it is necessary to apply the actual cautery, and to dress the wound with unguentum digestivum. A bougie of wax introduced into the opening, and the application of caustics, will in some cases produce the cicatrization of both the opening as well as the sac.

*Trichiasis* arises either from the eye-lids being turned inwardly to the eyes, the cause of which is not yet found out, or from the tarsus being turned inwardly, which may be occasioned by ulcers, scars, or a relaxation of that part. The cure of the first case is uncertain and defective. The author however effected a perfect cure in one case by making a small incision into the tarsus and cutting out a portion of the skin. If the tarsus be inverted, an incision in the skin near the affected part, and the cicatrix, thus produced, may in some cases be of great advantage.

The evacuation of blood produced by leeches is frequently insufficient in the *Chemosis*, in which case it is advisable to scarify the conjunctiva by means of a small pair of scissars, or to separate a part of the conjunctiva by a circular incision.

The simple *Macula Cornea* is but a slight superficial obscuration, produced by a chronic ophthalmia, which is preceded by a varixlike state of the veins in the conjunctiva and albuginea, or a dilatation of the small radicula of these veins on the surface of the cornea, whence a secretion of a transparent albuminous serum is produced in the substance of the conjunctiva, which covers the cornea. It is necessary to endeavour the contraction of the dilatated vessels, and to cut off their connection with the macula, which may be done by scarification; but the contraction of the vessels is best effected by the ointment of Janin.

*Albugo* and *Leucoma* are produced by an acute ophthalmia, in which a thick coagulable lymph passes into the substance of the cornea. A fresh albugo must be treated like the chemosis in the first and second period; but if the affection be old, such remedies are to be applied to the eye, which which promote the resorption. For this purpose Mr. Scarpa recommends: ℞. Aqu. calcis recent. unc. viij. sal. ammon. scrupl. ij. aerugin. gr. iv.; or the following ointment, ℞. Butyr. recent. unc. ß. tutiæ ppt. dr. ij. aloes, mercur. dulc. aa. gr. ij. The perforation of the leucoma and the production of an artificial ulcer in the leucoma are of little or no effect.

*Ulcers of the Cornea* are produced by the opening of a small abscess remaining after an acute ophthalmia, and such pustules should always be suffered to open by themselves. Ulcers of the cornea are of a livid colour, with thick irregular margins, painful, and secrete a corrosive matter. The best remedy is to touch them once or twice with lapis infernalis, till a cicatrization be produced. If a fungous excrescence arises in the ulcer it must be cut away with small scissars, together with the varices, after which the application of lapis infernalis is required.

*Pterygium,* is a preternatural membrane of a reddish livid colour and triangular figure, spreading from the internal cantus oculi towards the cornea; it consists of that part of the conjunctiva covering the cornea, which is degenerated, incrassated, and interwoven with varicous vessels. The triangular figure is owing to the conjunctiva adhering more strongly to the cornea towards the middle of it; the pterygium can be elevated in a fold. The cure of this complaint is to cut it away; sometimes a scar is produced, which obscures the cornea, but in a far less degree than the pterygium itself. Such pterygiums, which extend in the white of the eye, are separated only at the narrow part towards the end of the cornea, and cut away at their basis concentrically with the margin of the cornea. A tumour caruncula lacrymalis benignus is cured by the knife; but care must be taken not to cut away too much, lest an incurable running of tears should be occasioned. If the cicatrization does not take place, the part must be cautiously touched with lapis infernalis. The matter collected in the hypopyum is not real pus, but a coagulable lymph which exudes from the coats of the choroidea and uvea. The abatement of the ophthalmia will diminish the hypopyum.

Mr. Scarpa disapproves the incision of the cornea, as by the least wound of that part the inflammation may be increased, and the secretion of the matter augmented. In some cases, the collection of matter comes on so suddenly as to cause a great extension of the cameræ oculi; and it frequently happens that an exulceration, obscuration, and rupture of the cornea take place, through which the iris falls; all which is often attended with much danger to the life of the patient. In this case it is advisable to make an incision in the middle of the cornea, of the length of half a line; and having seized that part of the cornea where it is cut in with the forceps, to cut it off with a pair of scissars, so as to make an opening of the form of a lens, thro' which the matter, the lens crystallina, and, by degrees, the corpus vitreum, passes. After the operation the eye is to be covered with a cataplasm of bread and milk.

In the cure of a fresh prolapsus iridis, we must endeavour to diminish the irritability of the part which is fallen out, and to destroy so much of it that it may no longer distend the labia of the wound. For this purpose he recommends touching the iris with lapis infernalis or butyrum antimonii. The pains thus caused are diminished by luke warm milk. If the prolapsus be old, or the iris adheres to the lips of the wound, we may cut it away, and afterwards touch it with lapis infernalis. The prolapsus of the membrana propria of the aqueous humor rarely occurs; it appears as a small vesicle filled with water, passing through a wound or ulcer of the cornea. Sometimes it is a prolapsus of the corpus vitreum. It may be cut away with a small pair of scissars; or, if it be too small, it may be opened with a lancet or a needle, when the fluid will run out and the membranes retire. A prolapsus of the choroidea was cured by the author by touching the part with lapis infernalis.

Mr. Scarpa prefers the depression of the cataract, or the couching, to the extraction: 1. because it is a much easier operation; 2. it is adapted to every species of cataract; 3. it is less attended with dangerous accidents; and, 4. should the operation not succeed, it may be several times repeated on the same eye. The lens is not only pressed downwards by the needle, but also immersed into the corpus vitreum. Hence the patient must turn the eye, in order to promote the immersion, before the couching needle is extracted. It is likewise advisable to tear the anterior superfices of the capsula lentis, in order to prevent the obscuration of the capsula, in case it remains. The posterior part of the capsula is generally lacerated in the same manner; and if this should not be the case, an obscuration of this part is never so great as to hinder the sight. The lens which is depressed, gradually disappears, and is by degrees absorbed. For the operation, our author uses a couching needle and the hook of Pelletier. The first should be very fine, in order to hurt the eye as little as possible; which will generally prevent the rising of the lens and the ulceration of the membranes. For the purpose of destroying at the same time the capsula and depressing the lens with easiness, Mr. S. makes use of a fine needle, moderately bent at the point, which is convex on the back and cutting at both sides, and has a groove on the concave side. During the operation the other eye must be covered. The operator is seated higher than the patient, and the needle is introduced with the convex part of the hook turned towards the body of the operator, at a distance of one line from the cornea, and somewhat under the transverse diameter of the pupil; the needle must at the same time be bent with the handle a little downwards, to make the point easier penetrate into the eye. Then the convex part of the needle is brought on the superior margin of the lens, which is pressed a little downwards, and the hook of the needle immediately conducted between the corpus ciliare and the lens crystallina. The point of the needle being directed behind, it is pushed into the anterior part of the lens, and by a circular motion the capsula is there to be destroyed and the lens to be immersed into the corpus vitreum. In this situation the needle is held, in order to remove any flakes that may appear in the pupil; after which the needle being turned, it is extracted in the opposite direction to which it was brought in. If the anterior part of the capsula remain, which often occasions a secondary cataract, the needle is retracted from the lens, but applied to the part in the same manner as before.

In the operation of the soft or caseous cataract, the rupture which is made in the capsule must exactly correspond with the opening of the pupil in its natural extension; the hardest pieces must be cut through with the point of the needle, and the rest must be brought into the anterior chamber, where it will go down and be gradually absorbed. If in the secondary cataract, pieces of the capsule should obstruct the pupil without adhering to it, they must all be brought into the anterior chamber, where they will be by degrees absorbed. If the capsule adheres to the iris, the adhesion may be separated with the needle and brought into the anterior chamber; but care must be taken, as much as possible, not to touch the iris. After the operation the eye is covered with soft and dry linen, and must not be exposed to light for three days.

In the *pupilla clausa*, which sometimes follows the extraction traction and depression of the cataract, the author recommends to make an artificial pupil, a method which is founded on his opinion of the iris being a membrane different from the choroidea. The circumference of the iris is to be separated from the ligamentum ciliare without making an opening into the cornea, for which purpose a fine straight needle is to be introduced into the eye, at a distance of two lines from the cornea, and conducted on the superior margin of the iris which is turned towards the nose, where the ligamentum ciliare is perforated, and thus the iris is separated by pressing the needle downwards.

The artificial exulceration of the staphyloma is of no use at all, and should the staphyloma become prominent between the eyelids, it is much better to cut it away, by making a semicircular incision in the lower part, and afterwards seizing the section with a pair of pincers, and then finishing the cut. A part of the iris is generally cut away, when the lens and corpus vitreum fall out. On the fourth day an inflammation supervenes; the ensuing suppuration is to be promoted by emollient cataplasmas, and the patient is supplied with an artificial eye. The imperfect fresh amaurosis, particularly the periodical, arises frequently, per consensum, from the stomach and primæ viæ, against which the cure must be directed. The topical application of the vapours of ammonia is at the same time of service.

